# Fungicide consumption exacerbates the negative effects of a common gut parasite in bumble bee microcolonies

**DOI:** 10.1098/rsos.250225

**Published:** 2025-04-16

**Authors:** Emily Runnion, Ellen Klinger, James Strange, Frances Sivakoff

**Affiliations:** ^1^ Department of Evolution, Ecology, and Organismal Biology, The Ohio State University, Columbus, OH, USA; ^2^ Department of Entomology, The Ohio State University, Columbus, OH, USA

**Keywords:** bumble bee, microcolony, fungicide, *Bombus impatiens*, *Crithidia bombi*, qPCR to detect *Crithidia*, sublethal effects

## Abstract

Bumble bees face numerous environmental stressors, including gut-parasite infection and exposure to agricultural fungicides, which can negatively impact colony health. This study evaluates the interactive effects of these stressors on bumble bee (*Bombus impatiens*) microcolonies, focusing on colony development, worker survival and parasite infection dynamics. Our aim in evaluating these interactions was to determine if bees would experience synergistic negative health outcomes compared to single- stressor exposures. We reared 40 queenless bumble bee microcolonies, and treated them with either fungicide-contaminated pollen, inoculation with a gut parasite, both, or neither. Contrary to original expectations, we did not observe significant synergistic interactions between the two stressors; however, we found that consumption of fungicide was associated with higher likelihood of gut-parasite infection, and delayed recovery from infection. Fungicide consumption was also connected to smaller workers, and smaller male offspring. We also found that gut-parasite infection was correlated with decreased pollen consumption overall, decreased worker survival and fewer developed pupae. This study provides insights into the impacts of co-occurring stressors affecting bumble bees and emphasizes the importance of sublethal effects on pollinator health.

## 1. Introduction

Bumble bees (*Bombus* spp.) are declining in both density and richness at the global scale, threatening losses to biodiversity [1] and food security [2,3]. Bumble bee declines have been attributed to an array of stressors, including habitat loss and loss of floral resources [4,5], pesticide use [6–8], parasites and pathogens [7,9–11] and climate change [4,12]. Historically, scientific studies detailing the effects of these stressors focused on a single stressor, but in reality, bumble bees are likely to encounter multiple stressors simultaneously. Since stressors can have disproportionate negative impacts when experienced in combination [12–14], an understanding of the combined effects of stressors is imperative in our efforts to further bumble bee conservation.

Parasites have received considerable recent attention for their potential role in bumble bee declines, with a higher prevalence of infection in declining populations [7,15–17]. In the United States, one of the most common and widespread groups of bumble bee parasites are those from the genus *Crithidia* [18,19]. These parasites infect their hosts via ingestion, where they establish in the host’s gut, and spread to other hosts via horizontal transmission through contact with infected feces [20]. *Crithidia spp*. infection alone does not appear to be the cause of bumble bee declines in the United States and has low virulence under natural conditions [18,21]. However, this virulence is context-dependent, where infection increases host mortality under stressful conditions [22]. For example, Brown *et al*. [23] found that *Crithidia* infection led to a 50% reduction in survival of nutritionally stressed worker bees. Thus, to understand the impact of *Crithidia* on bumble bee populations, it is imperative to study virulence with comorbidities present.

Recent work suggests that exposure to fungicides, though not directly lethal at field-realistic rates, in combination with parasite infection, may have disproportionately negative effects on bumble bees. In a landscape-scale analysis, McArt *et al*. [7] identified fungicide usage as the strongest predictor of bumble bee species range contraction and found that greater fungicide use predicted the prevalence of the gut parasite, *Vairimorpha* (synonymous with *Nosema*) *bombi*, in declining species. Similarly, in honey bees, Pettis *et al*. [24] found that colonies that collected pollen contaminated with the fungicide pyraclostrobin were almost three times more likely to have a *Vairimorpha* infection. Fungicides also cause sub-lethal effects in bumble bees that would likely exacerbate parasite infections; fungicide exposure alters microbiome and gut function, which can influence immune health [25].

Here, we investigate the potential interactive effects of two common stressors, gut-parasite infection and fungicide consumption, on bumble bee microcolonies. We predicted that microcolonies exposed to combined stressors would experience negative synergistic health outcomes compared to control colonies, and that microcolonies exposed to a single stressor would have intermediate outcomes. We also investigated how fungicide consumption affected infection dynamics. While nutritional stress is known to increase the virulence of *Crithidia bombi* in bumble bees [23], the mechanism behind context-dependent virulence is unknown. Stressful conditions may cause individuals to be less tolerant of an infection, such that a given parasite load will result in higher virulence. Alternatively, bumble bees may become less resistant to infection, which will result in higher parasite loads over time. Both tolerance and resistance are strategies for hosts to defend against parasites [26], and reductions in either can have fitness consequences for bumble bee colonies. We predicted that fungicide consumption would reduce bees’ resistance to infection and increase parasite load in individuals as measured through insect frass samples.

## 2. Material and methods

### 2.1. Study system

The common eastern bumble bee (*Bombus impatiens* Cresson) is a stable species found in the eastern half of North America [15,27]. It is a generalist pollinator that is reared commercially for agricultural pollination services and is used in microcolony studies to evaluate the effects of stressors on bumble bee biology [28,29]. Microcolonies of bumble bees are comprised of 5−10 female workers, isolated from the queen, wherein one worker will establish dominance and begin laying haploid male eggs [28]. Maternal colonies used to establish microcolonies were purchased from Biobest Inc. (Plant Products USA, Westland, MI). Microcolonies were reared at The Ohio State University’s Rothenbuhler Honey Bee Research Lab, where rearing room conditions were kept between 30°C and 32°C and 50−70% relative humidity [28].

*Crithidia bombi* (hereafter *Crithidia*) is a ubiquitous trypanosomatid gut parasite that can affect the foraging behaviour [30], central nervous functionality [31] and overall colony fitness of infected bumble bees [20,32]. The live samples of *Crithidia* acquired for our experiment were sent to us by the lab of Dr. Rebecca Irwin, field-sourced from Massachusetts, USA.

The fungicide Pristine® is produced by BASF (Ludwigshafen, Germany), is comprised of 12.8% pyraclostrobin, 25.2% boscalid and 62% additives, and is approved for outdoor use in a variety of crop systems (Pristine Fungicide. EPA Reg. No. 7969−199). Pristine® is widely used in agricultural systems and has been demonstrated to occur at high doses in bumble bee-collected pollen and nectar [33]. Pristine has been shown numerous times to be associated with sublethal effects in honey bees [34–37]. Literature reviews for our previous work discovered that the active ingredients in Pristine are found in the field at doses above 600 million ppb, and we demonstrated that Pristine is associated with delayed emergence and smaller body size of males in bumble bee microcolonies [38].

We established 40 queenless microcolonies of *B. impatiens* workers between June 9th and July 17th of 2022. Each microcolony consisted of 5 callow female workers, which we marked upon emergence with a uniquely numbered tag (Opalith queen number set from Betterbee, Greenwich, NY). Queenless microcolonies of bumble bee workers produce only haploid male offspring. Each microcolony was allowed a 48 h adjustment period following establishment, and any worker who died during this time was replaced. Microcolonies were reared on either uncontaminated pollen or pollen contaminated with a field-realistic dose of Pristine®. Each microcolony was originally established with a fungicide-free 1g pollen patty, made from sterilized and uncontaminated honey bee-collected pollen, and 1−2 g of wax material from their natal colony. Following initial 48 h adjustment period, each microcolony was given either a +*Fungicide* or *-Fungicide* 1 g pollen patty every 48 h, based on assigned treatment. We weighed each pollen patty before and after placement in the microcolony, to record pollen consumption. We placed evaporative control pollen patties at equivalent locations within the room to account for water loss. We supplied bees within each microcolony *ad libitum* a 50/50 sucrose solution (see electronic supplementary materials for additional rearing information, electronic supplementary material, table S1).

### 2.2. Experimental treatments

To evaluate the effects of fungicide consumption and parasite infection, alone and in combination, on bumble bee microcolony growth and individual health, we established the following treatments. Starting 48 h after establishment, we fed half of our microcolonies (*n* = 20) 1 g pollen patties containing 15 000 ppb of the commercial fungicide Pristine® while the remaining microcolonies (*n* = 20) continued to receive untreated pollen. These pollen patties were replaced every 48 h, and we calculated pollen consumption following Runnion *et al*. [38]. Fourteen days after the establishment of each microcolony, we removed each worker from their microcolony and starved them for 1 h. We then randomly selected one worker from half of the fungicide-fed microcolonies (*n* = 10) and from half of the fungicide-free microcolonies (*n* = 10) and fed 1 µl of 50/50 sucrose solution-*Crithidia* inoculum at a concentration of 600 *Crithidia* cells/µl (see electronic supplementary methods for inoculation details). Individuals from microcolonies not fed inoculum were instead fed 1 µl of 50/50 sucrose solution. We recorded the identity of the inoculated bee and returned all workers to their microcolonies within 2 h of removal. This resulted in four stress treatments: control (*-Fungicide*, -*Crithidia*), fungicide only (+*Fungicide*, - *Crithidia*), parasite only (-*Fungicide*, +*Crithidia*) and combined (*+Fungicide*, +*Crithidia*). There were 10 replicates of each treatment, blocked by spatial location in the rearing room. All workers within a block were sisters, sourced from the same commercial queenright colony. To ensure that all workers within a block were sourced from the same queenright colony and that all workers within a microcolony had emerged within the same 24 h, we implemented a tiered microcolony establishment practice (see electronic supplementary material). Due to the tiered structure of our microcolony initiation, we had three rounds of inoculation.

### 2.3. Microcolony monitoring

Following treatment establishment, microcolonies were monitored daily for worker survival, time until first oviposition and time until first offspring emergence. We collected male offspring within 24 h of emergence from their pupal case, recorded their wet weights, then froze them at −20°C. Subsequently, we recorded male dry weights after placing them in a drying oven at 60°C for 72 h. We measured the length of the right-wing radial cell (a proxy for bumble bee size, [23]) in units of 0.04 mm using a DinoLite Digital Microscope (DinoLite, US). We calculated each male’s relative abdominal fat values, following methods described by Brown *et al*. [23].

We froze microcolonies at −20°C one week after the first male emerged. Of the 40 microcolonies, 11 did not have live males emerge. We determined the freeze dates for these microcolonies by taking the average freeze day for the other microcolonies +4 standard deviations (35.63+16.12 days). After freezing, we dissected each microcolony and recorded counts of total brood cells, honey pots, eggs and dead and live larvae and/or pupae. We differentiated live and dead brood by color and texture, as well as wing and eye deformation [39]. Workers that died during the experiment were removed, their wet weights were recorded, and they were placed in a −20°C freezer until further processing. At the conclusion of the experiment, we dried all workers at 60°C for 72 h and recorded their weights.

### 2.4. Parasite infection dynamics

Beginning 24 h after inoculation, we collected frass from all workers in the experiment for 14 days, in 24 h intervals. To collect frass, we removed each bee from its microcolony and placed it in a 5-dram vial for 30 min to produce frass. If the bee did not produce frass passively, we used a sterile glass stir rod to apply pressure to the bee’s abdomen to induce frass production. We collected frass from all 720 bees to ensure there was no contamination of *Crithidia* in the -*Crithidia* microcolonies, and to keep the effects of handling stress consistent for all treatments.

We extracted DNA from all frass samples following modified protocols from ‘Rapid Isolation of Mammalian DNA’ (see electronic supplementary material, methods, ‘DNA Extraction’) [40]. We stored DNA extracts at −20°C until used in qPCR. We conducted quantitative PCR using a Mastercycler® RealPlex2 (Eppendorf, Hamburg, Germany) for DNA extracted from frass samples. We used a hemocytometer to develop positive control standards by calculating known quantities of *Crithidia/*µl, resulting in an assay detection limit of 3.75 cells/µl. An individual was therefore considered infected only when it exceeded the assay detection limit. We checked PCR products for correct amplicon sizes with melting curve analysis. To ensure no cross-contamination of the parasite occurred between treatments, we preformed qPCR on DNA extracted from frass collected on days 1 and 14 from all bees in -*Crithidia* microcolonies. We tested the frass from every bee in microcolonies within the +*Crithidia* treatments for each day of the 14 day monitoring period.

To investigate the effects of fungicide consumption on *Crithidia* infection in each bee over the 14 day period, for all +*Crithidia* bees we analyzed likelihood of infection, level of infection over time and how originally inoculated bees differed from their sisters both within microcolonies and across treatments using statistical methods described below.

### 2.5. Statistical analysis

All analyses were completed in R [41]. The experiment was designed as a 2 × 2 factorial, and as such each of our initial models included the interaction between fungicide consumption and *Crithidia* inoculation. ANOVA values and likelihood ratio tests revealed that this interaction was never significant (*p* > 0.05), so we dropped the interaction terms from all subsequent models. We chose the final fixed effects for all models using backwards model selection with the drop1 function and likelihood ratio tests (table 1). For those response variables where we took multiple measurements within a microcolony (e.g. male health metrics), we included microcolony identity as a random effect. For continuous response variables, we used Shapiro-Wilk tests to determine if our data were normally distributed. When either fungicide consumption or *Crithidia* inoculation had significant effect, we used the emmeans function in the emmeans package [42] to obtain the means and standard deviations. We confirmed the fit of the final models by examining residual scatterplots and Q–Q plots. For effects on male emergence and size, and brood production, we excluded Block 5, as no oviposition activity ever occurred within this block.

**Table 1.** Statistical results of microcolony health metrics. Each response variable describes effects and behaviour measured by *Bombus impatiens* queenless microcolonies. All models run in R [41].

response	model	predictor	*X* ^2^	*Df*	*p*‐value
pollen consumption	lmer	fungicide consumption*Time^2^	5.74	2	0.06
		parasite infection*Time^2^	20.06	2	<0.001
		time^2^	279.33	2	<0.001
dose of fungicide consumed	lmer	treatment	0.95	1	0.33
		workers alive at the time of consumption	45.39	1	<0.001
		time	181.01	1	<0.001
worker survival over time	coxme	fungicide consumption	0.55	1	0.46
		parasite infection	4.56	1	0.03
		inoculation round	6.54	2	0.04
		original inoculate status	3.76	1	0.05
worker dry weights (log scale)	lmer	fungicide consumption	3.8	1	0.05
		parasite infection	11.65	1	<0.001
		spatial block	27.46	8	<0.001
male count	glm (negative binomial)	fungicide consumption	2.74	1	0.09
		parasite infection	0.34	1	0.54
		spatial block	58.43	8	<0.001
		workers alive at experiment end	18.94	1	<0.001
male radial cell (cubed)	lmer	fungicide consumption	5.88	1	0.02
		parasite infection	0.32	1	0.57
male relative fat (square root)	lmer	fungicide consumption	3.1	1	0.08
		parasite infection	2.65	1	0.1
		spatial block	24.97	7	<0.001
male dry weight	lmer	fungicide consumption	0.66	1	0.42
		parasite infection	3.11	1	0.08
		spatial block	17.08	7	0.02
		workers alive at experiment end	2.83	1	0.09
male emerge days	glmer (negative binomial)	fungicide consumption	2.13	1	0.14
		parasite infection	0.07	1	0.79
		workers alive at experiment end	7.73	1	<0.01
pupae	glm (poisson)	fungicide consumption	<0.01	1	0.95
		parasite infection	4.89	1	0.03
		workers alive at experiment end	51.87	1	<0.001
		spatial block	5.39	8	<0.001
dead pupae	glm (negative binomial)	fungicide consumption	1.97	1	0.16
		parasite infection	0.11	1	0.73
		workers alive at experiment end	4.5	1	0.03
		spatial block	16.82	8	0.03
live pupae	glm (poisson)	fungicide consumption	<0.01	1	0.96
		parasite infection	5.4	1	0.02
		workers alive at experiment end	58.63	1	<0.001
		spatial block	45.22	8	<0.001
larvae	glm (negative binomial)	fungicide consumption	0.64	1	0.42
		parasite infection	0.04	1	0.85
		workers alive at experiment end	39.13	1	<0.001
		spatial block	66.94	8	<0.001
dead larvae	glm (negative binomial)	fungicide consumption	0.07	1	0.79
		parasite infection	0.39	1	0.53
		workers alive at experiment end	4.49	1	0.03
live larvae	glm (negative binomial)	fungicide consumption	0.52	1	0.47
		parasite infection	<0.01	1	0.97
		workers alive at experiment end	44.4	1	<0.001
		spatial block	82.51	8	<0.001
eggs	glm (negative binomial)	fungicide consumption	0.56	1	0.45
		parasite infection	<0.01	1	0.93
		workers alive at experiment end	15.78	1	<0.001
		spatial block	21.89	8	<0.01
brood cell	glm (negative binomial)	fungicide consumption	3.07	12	0.08
		parasite infection	0.01	1	0.92
		workers alive at experiment end	92.83	1	<0.001
		spatial block	102.71	8	<0.001
honey pots	glm (poisson)	fungicide consumption	3.51	1	0.06
		parasite infection	1.2	1	0.27
		workers alive at experiment end	8.02	1	<0.01
		spatial block	27.49	8	<0.001

#### 2.5.1. Pollen consumption

Using a generalized linear mixed effects model, we calculated the average amount of pollen consumed, after accounting for water loss, per microcolony over 48 h intervals as a repeated measures analysis with time held as a quadratic predictor of pollen consumed, and the fixed effects of fungicide consumption and parasite infection. To account for potential dose-dependent effects, we also calculated the average ppb of Pristine consumed as the percent of the pollen ball consumed times the original 15 000 ppb of Pristine in the pollen ball. We analyzed differences in dose consumed between the +*Crithidia+Fungicide* and *-Crithidia-Fungicide* microcolonies, using a generalized linear mixed model with the fixed effects of treatment, workers alive in the microcolony, time, and the random effect of microcolony id.

#### 2.5.2. Worker size and survival

We analyzed worker dry weights using a generalized linear model using the lmer function from the lme4 package [43]. The original model contained the fixed effects of fungicide consumption, *Crithidia* inoculation, if the worker had been the originally inoculated bee, spatial block and the random effect of microcolony ID. To meet assumptions of normality, we transformed the response variable of worker dry weight by taking the natural log. We calculated worker mortality over the course of the experiment using a mixed effects Cox proportional hazards model with the function coxme in the package coxme [44], using the fixed effects of *Crithidia* infection, fungicide consumption and the infection round, and the random effect of microcolony ID.

#### 2.5.3. Brood production

We analyzed the counts of total pupae, live pupae and honey pots using a general linear model with a Poisson distribution in the package MASS [45]. To account for overdispersion, we analyzed the counts of dead pupae, total, live, dead larvae, eggs, total males emerged, male emergence time and total brood cells using a general linear model with a negative binomial distribution. The original effects included in each model were fungicide consumption, *Crithidia* infection, spatial block and count of workers who were alive when the microcolony was frozen.

#### 2.5.4. Male health metrics

We calculated male dry weight, radial cell length and relative fat using generalized linear mixed effects models with the fixed effects of fungicide consumption, *Crithidia* infection, number of workers alive until the microcolony was frozen, and spatial block. Prior to analysis, we transformed male radial cell length by cubing it and relative fat by taking the square root to normalize the response variables.

#### 2.5.5. *Crithidia* infection

For all models investigating the effects of fungicide consumption on *Crithidia* infection we used the original fixed effects of fungicide consumption, spatial block, inoculation round and if the bee was the original inoculate. We determined likelihood of infection in each bee as the probability of detecting *Crithidia* cells above the detection limit (3.75 cells/µl) using a general linear model with binomial distributions and a logit link function. We evaluated the time from inoculation to first detection above the detection limit and the count of days a bee tested above the detection limit, both modelled with a generalized linear model with a negative binomial distribution and the random effect of microcolony. We modelled the maximum level of infection per bee, transformed on the log scale to fit assumptions of normality, using a zero-inflated generalized linear mixed model (ZIG) in the package NBZIMM [46] with the offset of days until first infection was detected, and the random effect of microcolony.

To investigate the effects of fungicide consumption on *Crithidia* infection over time, we binned the infection monitoring period into three distinct categories: ‘Early’ (Days 1−4), ‘Middle’ (Days 5−9) and ‘Late’ (Days 10−14). We modelled the level of infection per bee over time, transformed on the log scale for normality, using a generalized linear mixed model (glmer), with the fixed effects of the interaction between fungicide consumption and time period, and the additional fixed effects of if the bee was the originally inoculated bee, the inoculation round, and the random effect of bee ID. We specified the model with a Gamma distribution and a log link function to account for the skewed nature of the infection data. Treating time as a discrete variable allowed us to see the trends in the data more clearly, given our relatively small sample size.

## 3. Results

Of the 40 microcolonies in which we recorded worker health and dry weights, male emergence time and size metrics, and overall brood production, 25 had males successfully emerge, and 32 had at least one brood cell upon colony dissection (electronic supplementary material, table S2). The average microcolony duration for all 40 microcolonies was 45.08 (±4.73) days. One block (6) produced brood cells, and had eggs observed upon colony dissection; however, no microcolony in this block had a male successfully emerge for the duration of the experiment. In Block 5, the control microcolony produced a single honey pot, however no other oviposition behaviour occurred within this block. Statistical results for explanatory variables found to significantly affect microcolony response variables can be found in table 1.

### 3.1. Stressors independently negatively affect bumble bee health metrics

**Figure 1.**
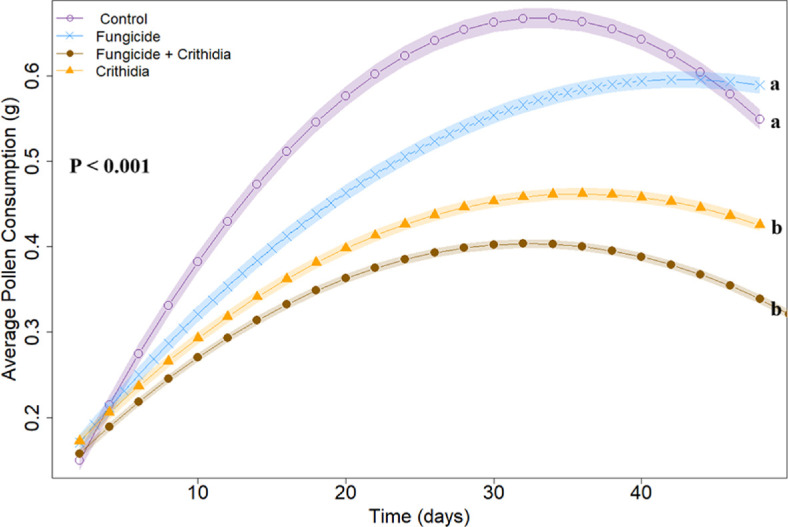
Parasite infection significantly reduced pollen consumption, regardless of fungicide contamination. Letters indicated a significant difference between curves. Transparent ribbons represent standard error, and lines designated by treatment-based symbols show the means over time. The most pollen, on average, was consistently consumed in the control group, without significant differentiation from the +*Fungicide* only group. Both treatments with +*Crithidia* consumed significantly less pollen on average, regardless of fungicide contamination of the pollen.

Microcolonies infected with *Crithidia* consumed significantly less pollen (*Χ*^2^ = 20.06, *p* < 0.001; figure 1), and, while not significant, microcolonies exposed to both stressors consumed the least pollen overall (mean = 0.38 g ± 0.05). The dose of fungicide did not vary between the +*Crithidia* and *-Crithidia* groups (*Χ*^2^ = 0.95, *p* = 0.33).

**Figure 2.**
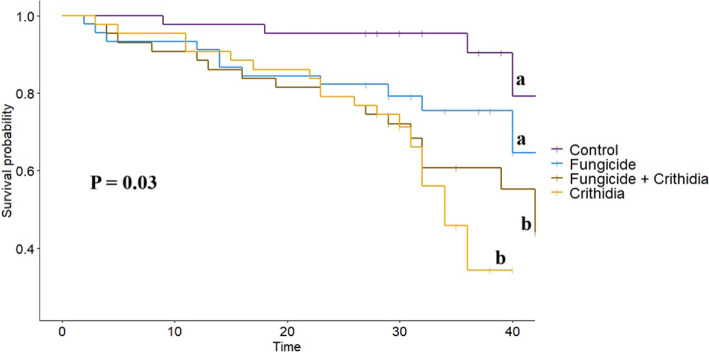
Cox proportional hazards analysis showing probability of worker survival across the duration of the experiment, for all four treatments. Letters deonte differences in groups, where ‘a’ is associated with *−Crithidia* groups and ‘b’ is associated +*Crithidia* groups. Parasite infection was associated with significant decrease in survival probability regardless of fungicide consumption.

**Figure 3.**
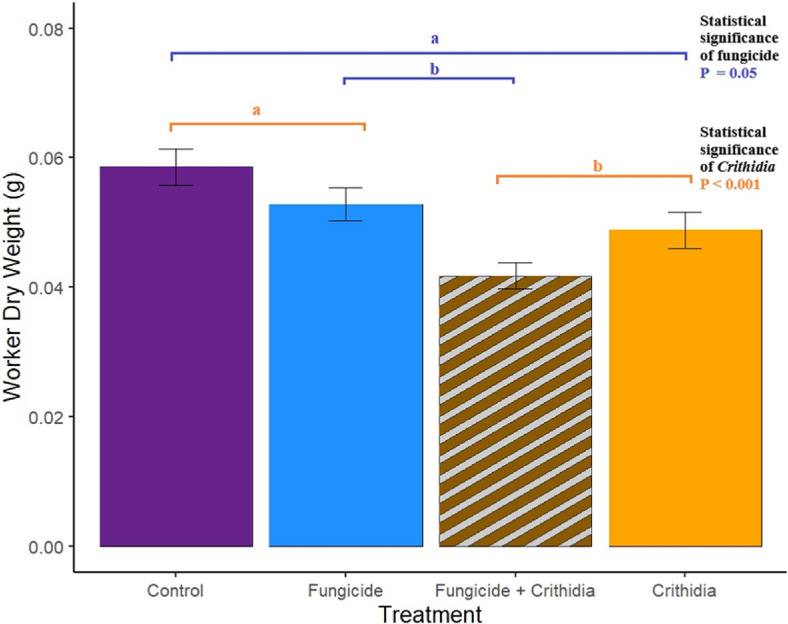
Both *Crithidia* and fungicide had negative effects on worker dry weights. Statistical significance of *Crithidia* is shown through letters in orange (‘a’ is associated with *−Crithidia* groups and ‘b’ is associated with +*Crithidia* groups), statistical significance of fungicide is shown through letters in blue (‘a’ is associated with *−Fungicide* groups and b is associated with +*Fungicide* groups). Average worker dry weights were significantly smaller in microcolonies exposed to both *Crithidia* and fungicide consumption, though model selection revealed no significant interaction between the stressors. As independent stressors, both *Crithidia* and fungicide were associated with decreased worker dry weights. Bars show standard errors.

**Figure 4.**
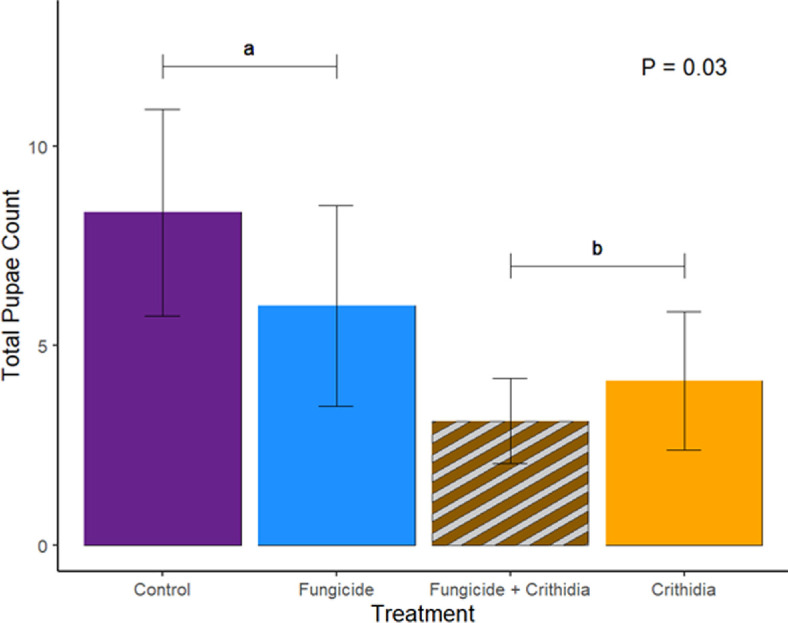
The average count of pupae per treatment based on dissection of the frozen microcolonies. *Crithidia* infection strongly predicted a decrease in total pupae count, but fungicide consumption was not meaningful in the model (*p* = 0.95). Letters signify significant differences in +*Crithidia (‘b’*) and *−Crithida (‘a’*) groups. Bars show standard error.

**Figure 5.**
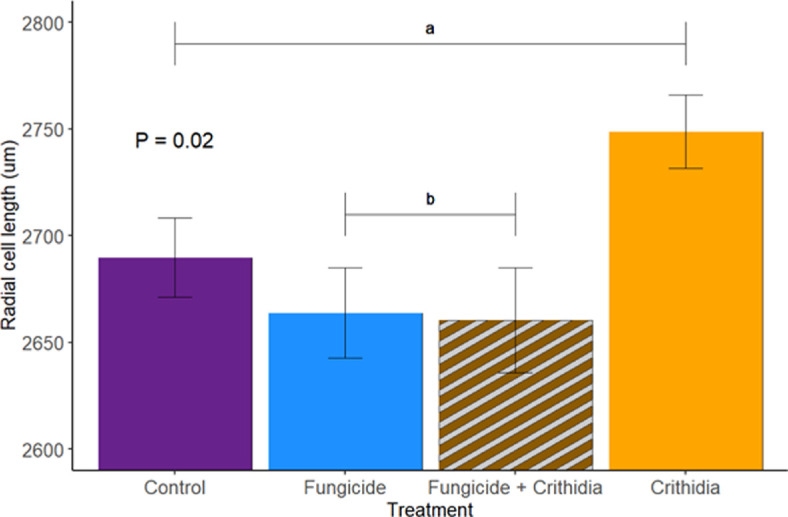
Males in microcolonies fed fungicide were significantly smaller, based on radial cell in wing measurements. Letters signify significant differences between the +*Fungicide (‘b’*) and *−Fungicide (‘a’*) groups. Bars show standard error.

Workers in microcolonies exposed to *Crithidia* had higher mortality by the end of the experiment (*Χ*^2^ = 13.83, *p* = 0.05; figure 2). While the interaction between *Crithidia* infection and fungicide consumption was not significant, both *Crithidia* infection (*Χ*^2^ = 11.65, *p* < 0.001) and fungicide consumption (*Χ*^2^ = 3.8, *p* = 0.05) independently were associated with smaller worker dry weights (figure 3). *Crithidia* infection was also associated with fewer total pupae (*Χ*^2^ = 4.89, *p* = 0.03; figure 4), as well as fewer live pupae counted within dissected microcolonies (*Χ*^2^ = 5.4, *p* = 0.02). Fungicide consumption did not correlate with altered male emergence, but did result in males with smaller radial cells (*Χ*^2^ = 5.88, *p* = 0.02; figure 5). Treatments of either fungicide consumption, *Crithidia* infection, or the combination had no significant effects on counts of larvae, eggs, honey pots, adult males, total brood cells or male relative abdominal fat. Microcolonies within all treatments had essentially no dead pupae observed upon dissection (electronic supplementary material, table S2).

In addition to our stress treatments, other explanatory variables affected our measured health metrics. Location in the rearing room had a significant effect on worker dry weights, the count of males, male relative abdominal fat, male dry weights, total pupae, dead pupae, eggs, honey pots and total brood cells (electronic supplementary material, figure S1). Additionally, the number of workers surviving until the end of the experiment had a significant positive effect on total counts of males, brood cells, honey pots, total larvae, live and dead larvae, total pupae and live and dead pupae.

### 3.2. Bumble bees more likely to test positive for *Crithidia* when fed fungicide

Of the 918 bumble bee frass samples tested for *Crithidia* with qPCR, 307 samples were positive for infection above the detection limit. Of these samples, 172 were from +*Fungicide +Crithidia* microcolonies, and 135 were from *−Fungicide +Crithidia* microcolonies. No bees from non*-Crithidia* colonies tested positive. Explanatory variables found to significantly affect *Crithidia* infection response variables can be found in table 2. In-text statistical results reported with standard errors, all levels of infection reported on the original scale.

**Table 2.** Statistical results of infection dynamics. Each response variable describes infection level of *Crithidia bombi* measured via qPCR in *Bombus impatiens* frass samples. All models run in R [41].

response	model	predictor	*X* ^*2*^	*Df*	*p*‐value
likelihood of infection	glm	fungicide consumption	7.66	1	<0.01
		spatial block	59.46	9	<0.001
		inoculation round	13.94	2	<0.001
		original Inoculate Status	102.63	1	<0.001
days from infection until first infection detected	glmer (negative binomial)	fungicide consumption	1	1	0.32
		spatial block	21	9	0.01
		inoculation round	12.5	2	<0.01
		original Inoculate Status	7.4	1	<0.01
maximum infection	lme.zig	fungicide consumption	2.4	1	0.12
		spatial block	7.94	1	<0.01
		original Inoculate Status	19.38	2	<0.001
number of days a worker was infected	glmer (negative binomial)	fungicide consumption	0.55	1	0.46
		original Inoculate Status	27.96	1	<0.001
change in infection level over time	glmer	fungicide consumption	1.94	1	0.16
		time period	4.15	2	0.13
		original Inoculate Status	13.55	1	<0.001
		inoculation round	31.45	2	<0.001
		fungicide consumption*time period	19.11	2	<0.001

**Figure 6.**
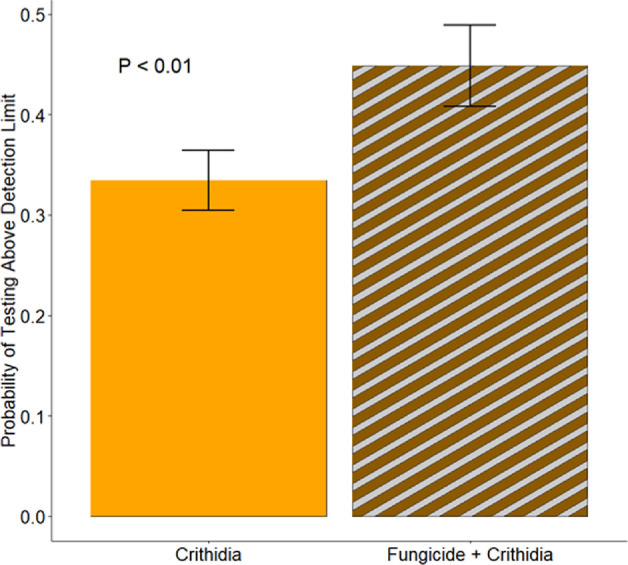
Workers in microcolonies fed fungicides were about 10% more likely to have an infection level detectable about the detection limit. Bars show standard error.

The average likelihood of infection level above the detection threshold was 10% higher in microcolonies that consumed fungicide (*Χ*^2^ = 7.66, *p* =<0.01; figure 6). Inoculation round was also significantly correlated with infection likelihood, where the last inoculated bees were most likely to be infected (40.8% ± 4%), followed by rounds 2 (23.2% ± 2%) and 1 (20.4% ± 2%).

The average time per microcolony from initial inoculation until first infection detected above the limit was not significantly impacted by fungicide consumption (*Χ*^2^ = 1, *p* = 0.32). It was correlated with inoculation round, where the final inoculated bees had the shortest time until first detection (0.59 days ± 0.27), followed by the second (2.53 days ± 0.94) and first (2.57 days ± 0.99) rounds of inoculation. The original inoculation status of the bee was also correlated with time until first detection, and the inoculated bees tested positive for infection one day before their non-inoculated sisters (2.03 days ± 0.39 versus 3.17 days ± 0.37, respectively).

The average maximum level of infection on the log-transformed scale per microcolony was not correlated with fungicide consumption (*Χ*^2^ = 2.4, *p* = 0.12). The original inoculate status of the bee was significantly associated with maximum infection, and originally inoculated bees had higher maximum infection levels than their non-inoculated sisters (106.02 cells/µl ± 18.36 versus 68.94 cells/µl ± 11.01, respectively). The total number of days a worker was infected was not significantly associated with fungicide consumption (*Χ*^2^ = 0.55, *p* = 0.46), but if the bee was the originally inoculated bee they were infected more often (6.29 days ± 1.3) than if they were not the original inoculate (2.01 days ± 0.31).

**Figure 7.**
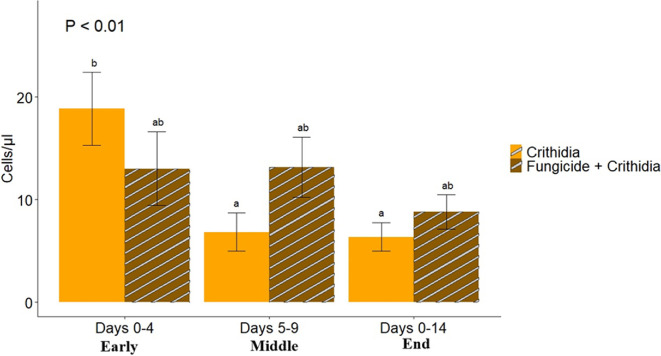
The change in average infection level is based on beginning, middle, and end of the 14 day frass monitoring period. Microcolonies that were fed fungicide-contaminated pollen did not ever significantly decrease their infection levels. Microcolonies that were not fed fungicide-contaminated pollen did decrease infection levels from the beginning to the end of the experiment. Letters signify significantly different groups and bars signify standard error.

We found a significant interaction between fungicide consumption and time period (*Χ*^2^ = 19.11, *p* < 0.001; figure 7). Only the bees that were not fed fungicide had a significant difference in the level of live *Crithidia* cells across the duration of time we collected frass, with an overall decrease in infection from beginning to end of the experiment. The original inoculate status had a significant positive effect on infection level (*Χ*^2^ = 28.19, *p* < 0.001), where the originally inoculated bees had higher overall infection levels (17.64 cells/µl ± 2.50) than did their non-inoculated sisters (9.22 cells/µl ± 1.16). The round of inoculation was significantly correlated with average infection level at all three time periods, where the third round had the highest overall average infection (29.93 cells/µl ± 4.68), followed by the second (10.55 cells/µl ± 2.08) and first (6.99 cells/µl ± 1.03) rounds.

## 4. Discussion

To our knowledge, this is the first study analyzing the combined effects of a fungicide and the gut-parasite *Crithidia bombi* on bumble bees. Contrary to our prediction, we do not find synergistic effects of the fungicide and the gut-parasite *Crithidia bombi* on *B. impatiens* microcolonies. Instead, both fungicide consumption and parasite infection affect microcolony development, but in different and potentially interconnected ways. While the stressors did not act synergistically, their individual effects could compound over time and lead to substantial sublethal impacts on bee health, colony dynamics and ecosystem services. We found that microcolonies with *Crithidia* infections had decreased pollen consumption, smaller workers, lowered worker survival and fewer pupae. Microcolonies fed fungicide-contaminated pollen produced smaller offspring overall. Fungicide consumption also increased the likelihood of parasite infection. The combination of these results has important implications for bumble bee colony health and pollination services.

We found that microcolonies exposed to *Crithidia* ate less pollen over time (figure 1). Interestingly, fungicide contamination of pollen did not significantly impact the quantity of pollen consumed, which aligns with our previous findings [38]. Pollen provides necessary proteins and lipids [47] and is imperative in the development of brood [48], decreased pollen consumption suggests that *Crithidia* infection could ultimately alter foraging behaviour. Previous work by Gegear *et al*. [49] has shown that infection with *Crithidia bombi* can alter bumble bee foraging efficiency by decreasing the ability to recognize rewarding floral traits. *Crithidia* infection can alter central nervous system functionality [31] or prompt a host immune response [50] that may explain parasite infection’s impacts on foraging. Interestingly, bees alter their foraging behaviour based on *Crithidia* infection with the apparent goal of seeking resources containing medicinal properties [10]. Our study provides further evidence that infection with a gut parasite is associated with altered nutritional uptake, which is critical for colony growth.

Decreased pollen consumption is also associated with fewer and smaller workers [51]. Our findings support this relationship, with lower worker survival and decreased worker dry weights in microcolonies infected with *Crithidia* (figures 2 and 3). Our analysis of worker dry weights is the only response variable that revealed significant effects of both stressors, with fungicide consumption also associated with smaller workers. Worker size is an important predictor of pollination and foraging behaviour, where smaller bees are less resilient to climate change [52], have smaller foraging ranges [53], and are less efficient pollinators overall [54,55]. Worker mortality, as observed in *Crithidia*-infected microcolonies, further compromises colony size, reproductive success and pollination services [56]. Worker mortality is associated with smaller gynes [57]. Worker size is also a predictor of the jobs completed by workers throughout the colony [58–60], which has important implications for successful brood rearing and performing pollination services when foraging. Given that the +*Crithidia* microcolonies also produced fewer pupae (figure 4), our findings provide concerning implications for bumble bee colony growth when faced with gut-parasite infection.

*Crithidia* infection did not significantly impact newly emerged male size. But males in +*Fungicide* microcolonies had smaller body sizes as measured by their radial cells (figure 5). It is likely that fungicide consumed through pollen will similarly decrease newly emerged worker size as well, though we could not directly measure that through this microcolony study. The combined effects of the two stressors on size, both of the parasite and fungicide on the adult workers and the fungicide on the emerging offspring, have important implications for reproductive success and processes. Larger males produce more sperm [61] and copulate more quickly [62]. The impacts of decreased worker size on colony health and reproduction are covered above. These findings suggest that fungicide consumption in conjunction with *Crithidia* infection may cause significant decreases in body size across all castes of a bumble bee colony.

Fungicide consumption was associated with an overall increased likelihood of infection (figure 6), and significantly influenced the levels of parasite infection in workers over time (figure 7). We did not see a difference in average infection level between *−Fungicide* and +*Fungicide* microcolonies; rather, the important difference was seen in the change in infection level over time. Workers in microcolonies not fed fungicide had significantly lower *Crithidia* cell counts in the Middle and Late time periods compared to the Early time period. In contrast, workers in microcolonies fed fungicide did not show a decrease in pathogen load over time. This suggests that fungicide consumption reduces bumble bees’ ability to clear gut-parasite infections. We saw important differences between the originally inoculated bees and their non-inoculated sisters. Regardless of fungicide consumption, originally inoculated bees did not decrease infection levels over time and their overall pathogen loads were higher than those of non-inoculated bees. This is perhaps because their infection source, the sugar water containing 6oo *Crithidia* cells, had more live cells than their sisters received via horizontal transmission within the microcolony. Otterstatter and Thomas [63] found that initial infectious dose was directly correlated with live cell count in feces, and artificially inoculated bees held higher pathogen loads, which supports the differences we see between our inoculated bees and their sisters. The implication of this is that the source and level of original infection are directly tied to the virulence of the parasite in an infected bee. While some pathogens may be highly virulent at low doses, others may be able to be tolerated at a high dose.

We used qPCR to detect and quantify *Crithidia* infections from bumble bee frass, which offers a non-destructive alternative to gut sampling. Measurements of the parasite in frass are inherently going to be lower than measurements of the gut, and in fact, Wolmuth-Gordon *et al*. [64] found that bumble bee feces contained less than half as many live cells as did a gut-counterpart sample. However, frass analysis allows repeated measurements of individual infections over time and likely reflects parasite levels encountered during horizontal transmission in the field, such as through fecal contamination at floral sites [65]. This is likely also true of horizontal transmission to nestmates within a colony, again making frass-based measurements a more realistic way to quantify the disease dynamics of the *Crithidia-Bombus* system. Traditional *Crithidia* studies often rely on hemocytometer-based gut quantification, which is less sensitive than molecular approaches like qPCR. In this study, we used a hemocytometer to measure the initial inoculum of live parasites fed to bees. However, inoculation round significantly influenced infection dynamics, highlighting the limitations of hemocytometer counts.

Our findings underscore the complex ways in which multiple stressors can cause both lethal and sublethal effects on bumble bee health. Fungicide exposure and *Crithidia* infection combined were shown here to have significant implications for bumble bee microcolony health but not direct mortality, and while we did not observe synergistic effects, both stressors independently compromised key aspects of colony development, reproduction, and survival. Additionally, exploring the mechanisms underlying altered foraging behaviour and nutritional uptake in infected bees could provide critical insights into colony-level resilience. These results emphasize the utility of non-destructive sampling methods and highlight the need for approaches that measure infection levels indicative of real-world horizontal transmission. By integrating molecular approaches with field-based studies, we can develop strategies to mitigate the compounded threats of agricultural practices and pathogens to pollinator populations. We show here that the combination of fungicide and *Crithidia* may not directly interact to affect bumble bee mortality, but the associated interactions of their sublethal effects may ultimately cascade to cause colony reductions.

## Data Availability

Data can be accessed via Dryad [66]. Supplementary material is available online [67].
